# Deficit of Inhibition as a Marker of Neuroplasticity (DEFINE Study) in Rehabilitation: A Longitudinal Cohort Study Protocol

**DOI:** 10.3389/fneur.2021.695406

**Published:** 2021-08-09

**Authors:** Marcel Simis, Marta Imamura, Paulo Sampaio de Melo, Anna Marduy, Linamara Battistella, Felipe Fregni

**Affiliations:** ^1^Núcleo de Estudos Avançados em Reabilitação, Universidade de São Paulo, São Paulo, Brazil; ^2^Neuromodulation Center and Center for Clinical Research Learning, Spaulding Rehabilitation Hospital, Boston, MA, United States

**Keywords:** disability, biomarkers, brain plasticity, stroke, spinal cord injury, amputation, osteoarthritis, neuronal plasticity

## Abstract

**Background:** Brain plasticity is an intrinsic property of the nervous system, which is modified during its lifetime. This is one mechanism of recuperation after injuries with an important role in rehabilitation. Evidence suggests that injuries in the nervous system disturb the stability between inhibition and excitability essential for the recuperation process of neuroplasticity. However, the mechanisms involved in this balance are not completely understood and, besides the advancement in the field, the knowledge has had a low impact on the rehabilitation practice. Therefore, the understanding of the relationship between biomarkers and functional disability may help to optimize and individualize treatments and build consistent studies in the future.

**Methods:** This cohort study, the deficit of inhibition as a marker of neuroplasticity study, will follow four groups (stroke, spinal cord injury, limb amputation, and osteoarthritis) to understand the neuroplasticity mechanisms involved in motor rehabilitation. We will recruit 500 subjects (including 100 age- and sex-matched controls). A battery of neurophysiological assessments, transcranial magnetic stimulation, electroencephalography, functional near-infrared spectroscopy, and magnetic resonance imaging, is going to be used to assess plasticity on the motor cortex before and after rehabilitation. One of the main hypotheses in this cohort is that the level of intracortical inhibition is related to functional deficits. We expect to develop a better understanding of the neuroplasticity mechanisms involved in the rehabilitation, and we expect to build neurophysiological “transdiagnostic” biomarkers, especially the markers of inhibition, which will have great relevance in the scientific and therapeutic improvement in rehabilitation. The relationship between neurophysiological and clinical outcomes will be analyzed using linear and logistic regression models.

**Discussion:** By evaluating the reliability of electroencephalography, functional near-infrared spectroscopy, transcranial magnetic stimulation, and magnetic resonance imaging measures as possible biomarkers for neurologic rehabilitation in different neurologic disorders, this study will aid in the understanding of brain plasticity mechanisms in rehabilitation, allowing more effective approaches and screening methods to take place.

## Introduction

The brain is designed and molded by environmental changes, pressures, and experiences (1). In this context, brain plasticity is understood as an intrinsic and permanent property of the nervous system, which is in constant modification during the human lifetime (1–3). This property allows the partial or total recovery after injuries to the human nervous system, and it involves recovery or compensation and motor adaptation with assistive technologies (3). Throughout life, the human brain is flexible, adapts quickly to environmental changes, and simultaneously, preserves a relatively stable balance between the long-term potentiation and the long-term depression or excitability and inhibition (4, 5). However, evidence suggests that injuries in the nervous system unbalance neural stability (6). For example, recently, evidence has shown that a deficit in neuronal inhibition is detected in patients with a disability regardless of etiology (7–9), and this lack of inhibition is associated with more disability (10). The understanding of brain plasticity has advanced; however, this knowledge has had a low impact on the rehabilitation practice (1). Furthermore, the mechanisms involved in the excitability and the inhibition balance and in brain plasticity regulation are not completely understood, as the biomarkers would be able to measure these processes. Also, there is a lack of studies designed to understand the mechanisms of brain plasticity in the context of rehabilitation, and there are no reliable biomarkers in the rehabilitation field nowadays. Therefore, this study's objective is to identify, through transcranial magnetic stimulation (TMS), electroencephalography (EEG), and functional near-infrared spectroscopy (fNIRS), biomarkers that represent the imbalance in the cerebral activity and its impact on rehabilitation, optimizing, and individualizing treatments and resources. Consequently, this project looks to understand the relationship between the biomarkers and the functional impairment related to the disability, regardless of the etiology. For that reason, we designed this cohort study with four conditions in neurorehabilitation: stroke, spinal cord injury, limb amputation, and knee osteoarthritis (OA). We selected these four conditions to represent conditions with different levels of neural lesions (i.e., stroke for central lesions, spinal cord injury to central lesion but in spinal cord, phantom limb pain for peripheral lesion but more proximal, and OA for peripheral lesion and more distal). Subjects included in this cohort are tested in the baseline with comprehensive neurophysiological assessments to assess different measures of neuroplasticity and especially inhibitory activity of the motor cortex and also a comprehensive clinical assessment to correlate to functional deficits.

## Methods and Analysis

### Participants and Study Design

This is a prospective cohort study made up of four groups with a specific diagnosis (stroke, spinal cord injury, knee OA, or lower limb amputation) and a group of healthy volunteers.

Patients admitted to the conventional rehabilitation program of the Instituto de Medicina Física e Reabilitação (IMREA) will be invited to participate in the study and included after signing the informed consent form previously approved by the Hospital das Clínicas da Faculdade de Medicina da Universidade de São Paulo Ethics Committee for Research Protocol Analysis CAAE: 86832518.7.0000.0068. For this study, 400 patients will be recruited, 100 patients with a stroke diagnosis, 100 with knee OA, 100 with spinal cord injury, and 100 amputee patients. The control group will be made up of 100 healthy subjects paired by sex and age.

Patients who agree to participate in the study will undergo a series of assessments at two time points: before and after the IMREA rehabilitation program. An individualized approach characterizes this program, considering the injury's etiology, the type of disabilities the patient has, general clinical conditions, likely prognosis, and the patient's socioeconomic factors. The control group will only perform neurophysiological (EEG, fNIRS, and TMS) and functional assessments once.

It is important to note that all patients involved in the research will receive the same type of treatment as patients who are not participating in the research.

### Sample Size

The sample size of 100 patients for each type of injury was determined, given this is an observational study (prospective cohort) and, thus, the primary analysis being a linear regression. Therefore, 100 participants in each group will allow for the modeling of 10 covariates, which will yield an effect size of ~0.3, which we believe is enough, mainly due to the use of neurophysiological variables and our knowledge of previous studies. Our group recently performed two studies with multivariate analyses, one with 35 and another with 55 patients, to assess neurophysiological markers (EEG and TMS), which allowed us to identify the role of these markers in the rehabilitation of conditions such as stroke.

Thus, using a larger sample (100 subjects), compared with our previous studies, associated with a larger detailing of clinical and neurophysiological information, will aid in the better comprehension of cerebral plasticity. We also believe this sample size will yield similar results for patients with knee OA, spinal cord injury, and amputation. Furthermore, it will allow for the identification of transdiagnostic markers, not aiming to compare the different injury groups but to determine the neurophysiological alterations they have in common.

### Inclusion Criteria

Participants of both sexes will be included in the study if they are older than 18 years, have confirmed clinical stability verified by medical evaluation, have signed the informed consent form, and if they fulfill the eligibility criteria for the IMREA rehabilitation program. To be included in the specific injury groups, patients will have to have a clinical, and radiological [magnetic resonance imaging (MRI) or computerized tomography; or bilateral knee radiography] diagnosis of stroke (confirmed by computed tomography scan and/or MRI), spinal cord injury, or knee OA (clinically confirmed) or a clinical diagnosis of bilateral or unilateral lower limb amputation.

### Exclusion Criteria

Subjects will be excluded if they are pregnant, have active OA with clinical manifestations in joints other than the knee, or if they have any other clinical or social conditions that interfere with the patient's participation in the rehabilitation treatment.

### Clinical and Functional Assessments

At the beginning of the study, on the participant's first visit, a trained physician will take the patient's history, perform a physical examination, and review the eligibility criteria for that patient. The obtained information from the patient's history such as age, sex, height, weight, ethnicity, education level, medications in current use, comorbidities, and the specific characteristics of each injury will be used as covariates in the final linear regression model.

Several instruments that allow the global assessment of participants, general or specific to each condition, were selected (Table 1 and Appendix 1). Some scales, such as cognitive, sleep, and mood scales, will be used to characterize the study's sample, as well as for the management of confounding variables on the multivariate statistical model.

**Table 1:** Assessment instruments used for each study group.

**Category**	**Instrument**	**Amp**.	**Stroke**	**OA**	**SCI**	CG^*^
Independence	Functional Independence Measure (FIM) (11)	X	X	X	X	X
Independence (Specific to SCI)	Spinal Cord Injury Independence Measure (SCIM III) (12)				X	
Cognition	Montreal Cognitive Assessment (MOCA) (13)	X	X	X	X	X
Cognition (speach)	Semantic verbal fluency test (14)		X			
Pain	Conditioned Pain Modulation (CPM) (15)	X	X	X	X	X
Pain	Pressure Pain Threshold (PPT) (16)	X	X	X	X	X
Pain/Sensitivity	Monofilament Sensitivity Test (17)	X	X	X	X	X
Pain/Sensitivity	Tuning Fork Vibration Sensitivity Test (18)	X	X	X	X	X
Pain	Pain Catastrophizing Scale (19, 20)	X	X	X	X	
Pain	McGill Pain Questionnaire (Brazilian Version) (21)	X	X	X	X	
Pain	Visual Analogue Scale (VAS) for Pain (22)	X	X	X	X	
Lower Limb Motor Function	6-min and 10 meters gait test (23)	X	X	X	X	X
Lower Limb Motor Function	Timed Up and Go (TUG) (24)	X	X	X	X	X
Lower Limb Motor Function	Lower Limb Isokinetic Dynamometer (25)	X	X	X	X	X
Lower Limb Motor Function	Walk Index for Spinal Cord Injury (WISCI-II) (26)				X	
Upper Limb Motor Function	Fugl–Meyer Assessment (FMA) (27)		X		X	
Upper Limb Motor Function	Hand Grip and Pinching (28–30)		X		X	X
Upper Limb Motor Function	Purdue Pegboard Test (PPBT) (31)		X		X	X
Upper Limb Motor Function	Robot-Measured Kinematic Variables (32)		X		X	X
Upper Limb Motor Function	Finger Tapping (FT) (33)		X		X	X
Upper and Lower Limb Motor Function	Medical Research Council Scale (MRC) (34)		X	X	X	
Balance	Force platform (35)	X	X	X	X	X
Balance	Berg Balance Scale (36)	X	X	X	X	
Spasticity	Modified Ashworth (37)		X		X	
Sleep	Epworth sleepiness scale (38)	X	X	X	X	X
Mood	Hamilton Depression Rating Scale (HAM-D) (39)	X	X	X	X	X
Mood	Hospital Anxiety and Depression Scale (HADS) (40)	X	X	X	X	X
Pain, rigidity, and daily activities	Western Ontario and McMaster Universities Osteoarthritis Index (WOMAC) (41)			X		
Lower limb	Amputee Mobility Predictor (AMP) (42)	X				
Amputation	Quebec User Evaluation of Satisfaction with Assistive Technology (QUEST 2.0) (43)	X				
Stroke	National Institutes of Health Stroke Scale (NIHSS) (44)		X			
SCI	American Spinal Injury Association Impairment Scale (ASIA) (45)				X	
OA	Kellgren-Lawrence Radiographic Classification of OA (46)			X		
OA	Ultrasound Assessment (US)			X		
Quality of life	Stroke Impact Scale (SIS) (47)		X			
Quality of life	Medical Outcomes Short-Form Health Survey (SF-36) (48)	X	X	X	X	X
Polymorphisms	Genetic Polymorphism Analysis (49)	X	X	X	X	X

* *Control group*.

The same evaluator will preferably carry out assessments. Evaluators will be trained to standardize questionnaire applications to reduce assessment subjectivity.

A detailed explanation of all the scales and tests used in this study under Supplementary Material.

### Neurophysiological Assessment Methods

#### Transcranial Magnetic Stimulation

The Magstim Rapid® stimulator (The Magstim Company Limited, United Kingdom) and a 70-mm coil in figure-of-eight will be used, positioned tangentially to the skull and at an angle of 45° in relation to the sagittal line will be used. The muscular response to the stimulus applied to the motor cortex will be recorded using surface electromyography with Ag/AgCl electrodes positioned on the target muscle and the grounding electrode positioned on the wrist. We will follow the methods established in the literature for physiological and clinical studies (50).

Bilateral upper limb assessment (first dorsal interosseous muscle of the hand) will be performed. The motor area corresponding to the first dorsal interosseous is the most used motor cortical area in cortical excitability studies in addition to presenting a greater accuracy of the method due to the local anatomy and penetration of the TMS pulse. To locate the cortical area of the hand, it will initially be identified from the vertex (intersection between the nasion–inion lines and zygomatic arches). Then, for the identification of the probable hot spot, a mark will be made 5 cm from the vertex toward the ear tragus in the coronal plane. The hot spot will be defined as the location as the lowest resting motor threshold and with the greatest amplitude of the motor evoked potential in the target muscle for a given intensity of the upper threshold stimulus.

The resting motor threshold (rMT) will be defined as the minimum intensity necessary for a single TMS pulse on the hot spot to generate an evoked motor potential (EMP), with at least 50 μV peak-to-peak amplitude, in 50% of attempts. rMT will be used as an indirect measure of cortical excitability. In addition, the following measures will be used: resting EMP, in which motor 10 EMPs will be recorded, with an interval of ~7 s between stimuli; silent period (SP), which represents the temporary suppression of electromyographic activity during a sustained EMP voluntary contraction; intercortical inhibition (SICI), which will be assessed by interstimulus intervals of 2 ms. The conditioned stimulus intensity (CS) will be set at 80% rMT and the test stimulus intensity (TS) adjusted to induce MEPs of ~1 mV peak-to-peak amplitude. Also, finally, intracortical facilitation (ICF) will also be measured by 10-ms interim stimulus intervals, and CS intensity will be the same as it was for the SICI evaluation.

#### Electroencephalography

EEG is a useful tool for inhibitory network assessment. This study aims to understand better the effects of inhibitory networks on the rehabilitation process. Successful inhibition is associated with increased EEG power in theta and delta bands (51, 52). Thus, we hypothesize that a better functional state will be related to the increase in power in the EEG delta and theta bands in the resting state, as well-modifications of others EEG bands (alpha, beta, and gamma).

We will use the same methodology of our other trial also looking at the inhibitory activity using EEG (53). EEG will take place over ~45 min: 25 min of participant and software preparation, 10 min of EEG recording divided into a resting EEG condition (5 min with eyes open and 5 min with eyes closed), and a task-related condition (8 min). Participants will be asked to relax in the resting condition; the investigator will ensure they do not fall asleep.

The task-related condition will include movement observation, movement imagery, and movement execution. This will be recorded by connecting the Net Station software (for EGI) with E-Prime. The entire task-related condition part will consist of 60 trials, with 20 trials for each of movement observation, movement imagery, and movement execution in a randomized order.

We will record the EEG in a standardized way using the 64-channel EGI system (EGI, Eugene, USA). The EEG will be recorded with a band-pass filter of 0.3–200 Hz and digitized at the sampling rate of 250 Hz by connecting the Net Station software (for EGI) with E-Prime.

#### Electroencephalography Data Assessment

The EEG data will initially be analyzed visually by a specialist clinical neurophysiologist who will identify and signal the artifacts, in addition to possible clinical changes in the EEG. Then, the data will be exported and analyzed offline with EEGLab (54) and MATLAB (MATLAB R2014b, The MathWorks Inc. Natick, MA, 2000). The following standard bands and frequencies will be analyzed: delta (2–4 Hz), theta (4–8 Hz), alpha 1 (8–10.5 Hz), alpha 2 (10.5–13 Hz), beta 1 (13–20 Hz), and beta 2 (20–30 Hz). Sensory inhibition will also be analyzed through the methods already described in this protocol.

Furthermore, a coherence analysis will be carried out through the MATLAB “mscohere” function, which calculates the estimate of coherence squared of magnitude, which is a function of power spectral density and the cross power spectral density of two channels. A coherence value between 0 and 1 will be calculated for each frequency point for the selected channel pairs. High coherence between two EEG signals has been considered as evidence of the possible existence of a structural and functional connection between two cortical areas.

Initially, only the electroencephalographic activities related to the primary motor cortex (CZ, C3, and C4) will be used for multivariate regression models, in addition to transversal inter-hemispheric coherence (C3–C4) and front-central intra-hemispheric coherence, F3–C3, and F4–C4. The data referring to the other EEG channels will be stored for future exploratory analyzes.

#### Functional Near-Infrared Spectroscopy

The relative changes in the concentration of oxy- and deoxyhemoglobin will be evaluated for each condition (left-hand grip, right-hand grip, and both hands grip), including rest (interval between each activity block). An average of the 10 trials will be made for each condition to improve the signal-to-noise ratio. A correlation analysis (seed-based correlation analysis) will also be carried out, estimating the strength of the neural connections related to the channels positioned on the M1 brain area, which can help elucidate the null hypothesis.

#### Functional Near-Infrared Spectroscopy + Electroencephalography Recording Protocol

EEG recording will be carried out concurrently with fNIRS. There will be 10 min of recording at rest (the first 5 min with the eyes closed and the last 5 min with the eyes open). Then, the patient will be asked to open and close his hands at a frequency of 1 Hz, using a video to guide the frequency of their movements. For fNIRS, the patient will be asked to open and close his hands at a frequency of 1 Hz, using a video to guide the frequency of their movements. There will be five 30-s blocks in which the patient will execute right-hand movements and five 30-s blocks imagining the right-hand movements, with an interval of 30 s between blocks. The same protocol will be repeated for the left hand and with simultaneous movements of both hands resulting in a total fNIRS collection time of ~30 min.

#### Magnetic Resonance Imaging

Diffusion tensor imaging with fractional anisotropy (FA) will be used to collect the MRIs. The regions of interest will be the bilateral primary motor cortices and fibers of the corpus callosum and corticospinal tract. After obtaining the diffusion tensor imaging data, FA values will be determined for all corpus callosum and corticospinal fibers. The FA value of 0.15 will be considered as a reliable threshold to isolate the white matter from the rest of the brain (55). The volumetric measurement of the motor cortex will also be taken.

Initially, only the primary motor cortex thickness will be used for the analysis. We will select 10% of the sample, resulting in a total of 40 patients. These results will be analyzed with multivariate models, including only these patients in a subgroup analysis.

### Statistical Analysis

For this analysis, we will use the multivariate regression model in which motor improvement will be the dependent variable and changes in inhibitory activity (resulting from neurophysiological assessments performed before and after treatment) will be the independent variables.

To control the impact of different conditions on the multivariate model, we will create a “dummy variable” for each disease etiology. Moreover, demographic characteristics (age, education, sex, and ethnicity) and clinically relevant characteristics (duration of illness, comorbidities, and the use of medications) will be tested, as well as specific aspects for each disease, such as the stroke side, the level of spinal cord injury, the degree of bone deformity of knee OA, and the level of lower limb amputation. The neurophysiological biomarkers described earlier will be tested in the same model.

Although this study's aim is not to test interventions but to identify changes in biomarkers related to functional improvement (regardless of the therapy performed), the different therapies performed by patients will be quantified, including information such as the number of sessions, frequency, duration, among others, which can be used in future analyses.

For secondary analyzes, functional improvement can be assessed by the several, general and specific, scales used depending on the evaluated disease. In this situation, we will use the calculation of the functional modification's effect size and not the absolute values of the scale for the analysis in the multivariate model.

Also, the motor function of the upper limb will only be assessed for patients with stroke and spinal cord injury, the main scale used being the Fugl–Meyer Assessment. In this case, an analysis similar to the one described earlier will be performed but only including these two populations.

Some of the scales, such as those for mood, pain, cognition, and sleep, will be used to characterize the sample, in addition to possibly being used to control confounders in the multivariate model. Besides, mood, pain, and cognitive disorder are commonly present in these populations, so we will perform an exploratory analysis using a similar statistic method but with scales related to these aspects as dependent variables.

In the statistical analysis for the results of the obtained polymorphisms, classical methods of case–control studies' epidemiological analysis will be applied. Odds ratio and the respective 95% confidence intervals will be estimated by unconditional logistic regression to simultaneously control potential confounding variables. To assess the relationship between the dependent variable (stroke and its subtypes) and the independent variables (polymorphisms of the evaluated genes, smoking, lipid variables, etc.), the statistical technique used will be logistic regression analysis, which allows the evaluation of disease risk associated with a given variable considering all other independent variables in the model.

## Discussion

This study will help understand the relationship between the brain plasticity biomarkers and functional disability not related to a specific etiology but related to central and peripheral neural injury. In addition, we expect that this study is going to build a better understanding of the brain modification associated with prosthesis adaptation and movement adaptation assisted technologies in patients with stroke and spinal cord injury. We also expect that we are going to be able to understand better the relationship between these biomarkers and motor deficit and other functional disabilities. Thus, the current challenge is to identify in human beings how multiple aspects of brain plasticity that happens in an integrated way influence the process of rehabilitation and to understand how different types of lesions in the peripheral and central nervous system modify the balance of brain plasticity.

We chose these four different conditions (stroke, spinal cord injury, OA, and amputation), as in all of them, we can find patients passing through maladaptive neuroplasticity regarding pain and disability (56–60). This approach may allow us to explore the possible transdiagnosis markers linked with plasticity in disability and pain regardless of the diagnosis label usually touched by the literature. One of the justifications that aim to explore these biomarkers is that chronic pain and disability conditions may share similar maladaptive changes regarding neuronal plasticity (61, 62).

In this study, we are looking to test and explore the neural inhibition through TMS, EEG, fNIRS, and MRI that have an impact on the rehabilitation clinical practice. We will also classify biomarkers as follows: (i) substitute, which indicates the biomarkers are modified with the “successful” rehabilitation; (ii) prognosis, which indicates the biomarkers indicate functional recovery regardless of rehabilitation therapy; and (iii) predictive, which indicates the biomarkers predict treatment response. We will investigate the activity of theta, delta, and beta waves on EEG, rMT, EMP, SICI, ICF, CS, and SP on TMS, the brain metabolic activity on fNIRS, and the motor cortex's volumetric mass on MRI to assess these aspects as potential biomarkers, characterizing them as either substitute, prognostic, or predictive (63).

The development of (i) substitute results for functional recovery and the understanding of the factors that influence the rehabilitation process will be possible to develop consistent and feasible studies. Currently, the majority of the studies in this field have low statistical power, without control of confounding variables, and reproducibility problems (64). In addition, the lack of substitute markers has allowed the approval of therapies without the background of consistent methodological studies. These distortions are not harmless and overwhelm the health system.

Another biomarker that we expect to identify is (ii) prognostic. The current models to determine the functional prognosis of patients with stroke or spinal cord injury are not accurate, making the therapeutic approach difficult. These biomarkers could specify the patient's recovery potential, providing earlier hospital discharges and avoiding unnecessary treatments. One example is a pain biomarker in spinal cord injury using intracortical inhibition. Studies have shown that subjects with spinal cord injury and pain have decreased intracortical inhibition (65, 66).

The final biomarker that we aim to identify is (iii) predictive of functional recovery. In this case, we are going to identify the plastic brain modifications that are related to functional recovery and that are induced by rehabilitation therapy (67). This understanding will allow the individualization of the treatment and potentialize future insights about the new approaches of rehabilitation.

To our knowledge, there is a lack of studies designed to understand brain plasticity in the rehabilitation context, and there is too little knowledge in the mechanisms involved in the balance between neural inhibition and excitability and in brain plasticity regulation. Also, the current literature has had a low impact on the rehabilitation practice.

In this context, TMS is a widely used tool to measure corticomotor excitability in patients with motor deficit conditions such as stroke. It has been found that motor deficit severity is highly associated with the level of corticomotor excitability measured by rMT depicted in TMS results. Given that rMT reflects neuronal membrane excitability, functional modifications that occur after motor deficit conditions, such as inhibitory circuit malfunctions, are reflected as rMT alterations in TMS (68). We expect to find a reduction in inhibitory brain activity associated with a decrease in rMT, SICI, and SP due to the direct relationship between these biomarkers and motor inhibitory pathways of the brain. Moreover, study findings show that ICF and MEP are mediated by different interneuronal pathways than those of rMT, SICI, and SP; thus, it is expected that as inhibitory circuits increase their activities, ICF and MEP amplitudes will be decreased (69).

Several studies have conveyed brain plasticity alterations in the primary motor cortex. These changes have been thus identified through the use of biomarkers such as EEG and fNIRS, for instance, strong correlations between theta and delta band activities in the hippocampus and successful inhibitory mechanisms in rodent models (70). The use of EEG as a biomarker “tracker” thus becomes important because sensory inhibition reflects an automatic cortical inhibition function, which can be used as a measure of the brain's inhibitory status (71–74). These findings support one of the study's main hypotheses that motor function deficits are associated with lower cerebral inhibitory activity when compared with a healthy control group. Studies that have performed electrophysiological evaluations of patients with chronic pain conditions such as OA, spinal cord injury, and neurogenic pain have found a decrease in theta and delta wave activity and an increase in beta wave activity due to deficient brain inhibitory circuits (75). Thus, an increase in the activity of theta and delta bands, as well as a decrease in beta bands, is a reflection of increased inhibitory activity, which can be achieved through rehabilitation in patients with pain conditions. Therefore, given these findings, we expect a diminished event-related desynchronization in patients with motor dysfunctions.

The association of EEG and fNIRS have been used in many studies to analyze cortical activation and plasticity connections in the context of rehabilitation (76). Both tools are practical, non-invasive methods of measuring brain activity; however, fNIRS lacks good spatial and temporal resolution and cannot assess deep brain structures (75). Thus, its association with EEG provides a more holistic understanding of cortical activation in the conditions being studied. Moreover, it has been shown that electrophysiological variations have the ability to predict hemodynamic activation in motor regions of the brain (77).

Different studies have found an inverse relationship between fNIRS results and efficient inhibitory networks, given that efficient inhibitory networks depict less cortical activation in prefrontal regions during executive function tasks. Therefore, we hypothesize that, as patients undergo rehabilitation, their inhibitory networks will be strengthened, which will then be depicted on the fNIRS as a decrease in activity, bolstering the use of fNIRS as a tool for plasticity biomarker measurement (78). However, evidence on this relationship is still conflicting. For instance, a study evaluating the feasibility of fNIRS as an assessment mechanism for patients with spinal cord injury has conveyed enhanced activation of motor cortical areas after rehabilitation with robot-assisted gait training (78). Given that fNIRS produces blood-oxygen-level-dependent images, this suggests a link between the brain's metabolic activity in the motor cortex and recovery of gait-impairing conditions such as SCI and stroke, which is still unclear. Thus, the use of fNIRS to evaluate neuroplasticity biomarkers in this study will help to further elucidate the relationship between neuroplasticity and brain hemodynamic activity.

Moreover, the use of more conventional imaging methods, such as MRI to evaluate the transdiagnostic nature of plasticity biomarkers, has become very promising given the association between cortical thickness and chronic pain (79, 80), amputations (81, 82), spinal cord injury (83, 84), and stroke (85, 86). These correlations support the analysis of volumetric measurements of the motor cortex to yield a better understanding of this relationship.

This is a novel and feasible observational study that will help the understanding, improvement, and development of the rehabilitation field and allows for the identification of several biomarkers at once, providing further insight for clinical practice in rehabilitation. Furthermore, this study was designed to answer our hypothesis in an optimized way, with a rigorous methodology. Besides that, one of the limitations is that our results will not provide a causal relationship between neurophysiological markers and functional and clinical outcomes. Also, the study may be underpowered, as there are four groups of intervention; to minimize that, we performed a sample size calculation based on the expected effect size regarding previous studies; the neurophysiological surrogate variables are something that could help to address this limitation also. The differences between severities of conditions could lead to different dropout rates in one of the more severe group conditions; this may unbalance the groups' size. Another limitation is that the variability of the clinical phenotypes of the conditions approached by this study could generate some noise in our results. To minimize this limitation, we are planning to do a multivariate analysis, which could minimize relevant confounders that may interfere with our results. Finally, our study may lack generalizability, as the study will be performed in only one center.

On the other hand, this is a novel and feasible observational study that, as mentioned before, will help the understanding, improvement, and development of the rehabilitation field.

In conclusion, understanding brain plasticity in the context of rehabilitation allows for more effective approaches and provides screening evaluations of current rehabilitation techniques. The future results of this study will help to better understand brain plasticity and its mechanisms and its reliable representative biomarkers as well. This knowledge will lead to the development of rehabilitation as has occurred in other fields, such as cardiology. In this context, simple questions such as the posology of the treatment will start to be answered when we have markers to measure the functional recovery and feasible and powered studies will be able to be performed in an extensive and consistent manner.

## Ethics Statement

The studies involving human participants were reviewed and approved by the ethics committee of Medical School of University of São Paulo (CAPPESQ). The patients/participants provided their written informed consent to participate in this study.

## Author Contributions

In this study protocol, FF conceived the initial idea, MS, MI, and LB designed the study, and PM, AM, MS, and FF drafted the manuscript article. All authors reviewed and approved the final version of the paper.

## Conflict of Interest

The authors declare that the research was conducted in the absence of any commercial or financial relationships that could be construed as a potential conflict of interest.

## Publisher's Note

All claims expressed in this article are solely those of the authors and do not necessarily represent those of their affiliated organizations, or those of the publisher, the editors and the reviewers. Any product that may be evaluated in this article, or claim that may be made by its manufacturer, is not guaranteed or endorsed by the publisher.
