# Supplementary material for: Deficit of Inhibition as a Marker of Neuroplasticity (DEFINE Study) in Rehabilitation: A Longitudinal Cohort Study Protocol
Source: Front Neurol. 2021 Aug 9;12:695406. doi: 10.3389/fneur.2021.695406 (PMC8380986; doi:10.3389/fneur.2021.695406)
Supplement: Supplementary file 1 [file Data_Sheet_1.pdf]

APPENDIX

Appendix 1. Functional Clinical Assessment Description and Application

Functional Independence Measure (FIM): This questionnaire evaluates the need for others' assistance a patient has to carry out the motor and cognitive functions (i.e., self-care, locomotion, sphincter control, communication and social cognition – memory, social interaction, and problem-solving). Score varies from 1 (completely dependent) to 7 (completely independent) [11].

Spinal Cord Injury Independence Measure (SCIM III): The third version of a spinal cord injury-specific scale used to assess the patient's independence and ability to perform tasks in the fields of self-care, breathing and sphincter control, and mobility. Score varies from 0 to 100 and is divided into the three evaluated fields (self-care: 0-20; breathing and sphincter control: 0-40; mobility: 0-40) [12].

Montreal Cognitive Assessment (MOCA): Screens the patient's dominance over their cognitive function's executive function, visuo-spacial ability, memory, attention, concentration, occupational memory, language, and temporal and spatial orientation. The scale has a maximum score of 30 points and an application time of approximately 10 minutes [13].

Category fluency test: The test is performed by asking the patient to say as many animals as possible in 1 minute. It is considered normal for the patient to say the name of nine or more animals, for individuals with up to eight years of incomplete schooling, and for the group with eight or more years of schooling, 13 animals is considered normal [14].

Conditioned pain modulation (CPM): Through intense heterotopic stimulation, this test evaluates a patient's endogenous modulation response to pain. Subjects will immerse one of their hands into a recipient containing cold water (10-12°C) for one minute. After 30 seconds of immersion, the Visual Analogue Scale (VAS) will be presented to patients so that they can indicate their pain level, referring to the submerged hand. Subsequently, three algometric measures will be taken (spaced between 15 seconds) for the contralateral hand. After an interval of approximately 10 minutes (time for hand to return to normal body temperature), the other hand will be immersed in the recipient, and follow the previously stated protocol [15].

Pressure Pain Threshold (PPT): This test uses an algometer to define the minimum amount of pressure that triggers pain in pre-established regions (thenar region, medium deltoid, and region located one inch above the patella) [16].

Monofilament Sensitivity Test: This test uses an esthesiometer with different width monofilaments to define patients' superficial sensibility and pain thresholds. Forces escalate through steps, reflecting the most critical functional thresholds of the hands and feet [17].

Tuning Fork Vibration Sensitivity Test: Validated way of measuring profound/deep sensitivity. Measures vibratory sensitivity and is tested on the bony prominences on the four limbs [18].

Pain Catastrophizing Scale: Made up of nine items staggered on a Likert scale, varying from 0-5 points related to the words "almost never" and "almost always" on the extremities. The total score

is the sum of the items divided by the number of answered items, with the minimum achievable score being 0 and the maximum 5. Higher scores indicate the presence of catastrophizing thoughts [19,20].

McGill Pain Questionnaire (Brazilian Version): A self-reported questionnaire that evaluates the quality and intensity of subjective pain. It is made up of 78 words, divided into four categories (pain descriptors, pain affective component, pain assessment, and miscellaneous). Subjects choose the words that best describe their experience with pain. They can be classified on an intensity scale from 0 (none), 1 (mild), 2 (moderate), or 3 (severe) [21].

Visual Analogue Scale (VAS) for Pain: Made up of a 10 cm straight line on a piece of paper. Written on one of its ends is the phrase "no pain" and on the other "maximum pain". Each subject will be asked to mark their discomfort level on the VAS line. The closer the mark is to the scale's origin (zero centimeters), the lower the patient's pain level. On the other hand, the closer the mark is to the scale's end (ten centimeters), the higher the patient's pain level. Instructions for the patient will be "Identify the amount of pain experienced in the last 48h and make a mark perpendicular to the 'no pain' – 'maximum pain' line" [22].

6-minute and 10 meters gait test [23]:

10-meter gait test: aims to assess a patient's short-distance walk speed. It is recommended that the subject walks 14 meters so that the 2 initial and final meters be disregarded. The subject will walk at their normal speed.

6-minute gait test: evaluates the maximum distance a subject can walk on a plane, rigid surface in six minutes through a 30-meter track. It is recommended that this be a 30-meter walk, lapped every 3 meters in which turning points are set with a cone.

Timed Up and Go (TUG): This test evaluates an individual's mobility level, measuring the amount of time it takes for the subject to stand up from a chair without using their arms, walk a 3-meter distance, turn 180° and get back to sit on the chair [24].

Lower Limb Isokinetic Dynamometer: Allows lower limb assessment, rehabilitation, and muscular training. Isokinetic evaluation allows for a better comprehension of muscle ability, providing parameters such as strength, effort, power, and the strength relationship between agonist and antagonist muscle groups. With this assessment, it is possible to perform bilateral comparisons, possible identification of muscle group deficits, and treatment and rehabilitation progression [25].

Walk Index for Spinal Cord Injury (WISCI-II): Functional ability scale used to measure march alterations in patients with SCI through the amount of physical or device assistance needed for them to walk 10 meters. The score varies from 0 (unable to stand up and/or walk with assistance) to 20 (walks 10 meters without any device assistance, orthoses, and physical assistance) [26].

Fugl-Meyer Assessment (FMA): Specifically evaluates the recovery of hemiplegic patients. The assessment is divided into 5 domains: motor function, sensitivity, balance, movement amplitude, and pain [27].

Hand grip and pinch: aims to evaluate muscle strength with a higher sensitivity than the MRC since it uses a continuous measure with a kg-strength scale measured by a dynamometer specific for hand grip and another for pinching.
The hand grip is a dynamometric measure of the maximum amount of strength produced during a five-finger squeeze. Three trials on each hand will be done to compute the average strength: the subject will be seated with arms by their torso and elbows flexed at 90 degrees, neutral forearm, and slightly extended pulse. If subjects cannot maintain the testing position, the dynamometer will be supported, with measures taken to ensure that this support won't interfere with the evaluation [28,29,30].

Purdue Pegboard Test (PPBT): aims to measure the speed and coordination of movements. The test is done with the elbow supported on the table for the assessment of the upper limb's distal coordination. For this evaluation, the time that it takes for the patient to put pins into the holes of a wooden board is measured. This test evaluates dexterity and coordination. It is performed on a wooden board made up of 2 sets of 25 holes (vertically arranged) and four concave holes on the board's superior part. The participant is asked to remove the pins from the concave holes and insert them on the vertical holes as quickly as possible. The number of pins placed in 30 seconds is scored [31].

Robotic Measurement of Kinematic Variables: robotic evaluation will obtain data about speed, acceleration, and joint angle, the time between each point, task execution time, and target displacement. This assessment is based on the evaluation protocol of the InMotion Arm© device, made up of different visually guided tasks in which the patient performs reach and circular movements and movement attempts against resistance. From this movement, variables such as deviation from a straight line, distance from the target, average speed, maximum speed, movement duration, shoulder joint strength, among others are obtained [32].

Finger Tapping (FT): This test measures the execution speed of simple movements. The patient is asked to turn on a lever on a wooden board, as many times as possible, using only their index finger, during 30 seconds. The assessment is performed on both hands, separately [33].

Medical Research Council Scale (MRC): It is an assessment performed quickly that classifies muscle strength in 5 different degrees. For the upper limb, shoulder abduction, elbow flexion and extension, and wrist and finger extension will be evaluated. As for the lower limb, knee extension and flexion will be evaluated [34].

Force platform: The force platform consists of two rigid surfaces, one upper and one lower, interconnected by force sensors. The most common rectangular force platforms on the market measure the three components of ground reaction force (GRF); each of the four force sensors records the force applied in the mid-lateral (X), anteroposterior (Y), and vertical (Z) directions. In addition to measuring the GRF components, it is also possible to obtain the Pressure Center (PC) [35].

Berg Balance Scale: this scale is made up of 14 tasks that assess static and dynamic balance, through tasks such as reaching, turning, transferring, standing, and standing up. The score for each

item ranges from 0-4, determined by the ability to perform the task, and the maximum score is 56 points [36].

Modified Ashworth Scale: used to assess spasticity, which is classified into 5 categories ranging from normal to stiffness, according to muscle resistance against passive movement. For the upper limb, shoulder abduction, elbow flexion and extension, and wrist and finger extension will be evaluated. As for the lower limb, knee extension and flexion will be evaluated [37].

Epworth sleepiness scale: Evaluates the degree of daytime sleepiness. It is a self-applied questionnaire that evaluates the probability of falling asleep in 8 everyday situations [38].

Hamilton Depression Rating Scale (HAM-D): The instrument investigates how the patient has been feeling in the last seven days, including the day of application; it consists of 17 items, which can be scored on a Likert scale ranging from 0 to 2 or 0 to 4, depending on the intensity of the symptom. The total number of points varies between 0 and 52 points. To verify the presence of depression, the scores must add up to at least 8 points in the original version [39].

Hospital Anxiety and Depression Scale (HADS): A 14-item scale that quantifies and qualifies symptoms of anxiety and depression. The scale contains 14 multiple-choice questions. It consists of two subscales, one for anxiety and another for depression, with seven items each. The global score in each subscale ranges from 0 to 21. It is intended to detect mild degrees of affective disorders in non-psychiatric environments. The patient is asked to respond based on how he felt during the last week [40].

Western Ontario and McMaster Universities Osteoarthritis Index (WOMAC): Scale used to measure pain, stiffness, and function in patients with knee and hip osteoarthritis. The questionnaire consists of 24 items, divided into 3 subscales: pain, addressing 5 items, stiffness, 2 items, and function, 17 items [41].

Amputee Mobility Predictor (AMP): evaluates the potential of gait with prostheses and can also be used to assess function during and after rehabilitation. The assessment consists of 21 items, with a score ranging from 0 to 42 [42].

Quebec User Evaluation of Satisfaction with Assistive Technology (QUEST 2.0): aims to assess patient satisfaction with different assistive technologies. This test consists of 12 items, eight of which refer to the criteria: dimension (size), weight, adjustments, safety, durability, simplicity of use, comfort, and effectiveness; and four items, referring to the service, including the criteria: delivery service, device repair, and maintenance, professional service and follow-up. The patient evaluates the device on a five-point scale that ranges from "not satisfied" to "very satisfied". Finally, patients identify the three most important items related to the evaluated device [43].

National Institutes of Health Stroke Scale (NIHSS): a scale composed of a 15-item neurological exam to evaluate consciousness level impairment, loss of visual field, language, neglect, eye movements, muscle strength, ataxia, dysarthria, and sensory loss in patients with acute stroke [44].

American Spinal Injury Association Impairment Scale (ASIA) (ASIA, 2003): a scale used to classify injury severity in individuals with spinal cord injury. It is a 5-point scale that classifies individuals from A (complete) to E (normal). The final score includes the sum of sensory and motor scores, in addition to sensory and motor anal assessment [45].

Kellgren-Lawrence Radiographic Classification of OA: it is a method of classifying the severity of knee osteoarthritis (OA), which uses five degrees: 0 (without osteoarthritis) to 4 (large osteophyte, marked narrowing of the joint space, severe sclerosis, and definite deformity of bony extremities) [46].

Ultrasound Assessment (US): This assessment will be performed bilaterally in the knee region at the baseline of patients with OA. The US will be used to detail the OA pathology itself and other associated pathologies.

Stroke Impact Scale (SIS): assesses the quality of life after the stroke. It is a self-reported questionnaire that aims to assess the impact of stroke in different domains. The questionnaire is arranged into 8 domains, in which the subject responds to questions on a 5-point Likert scale, based on the difficulty the patient has to perform each task. An extra question, on a scale of 0-100, is presented to the patient so that he can respond with his feeling of recovery after stroke. Test domains include strength, hand function, day-to-day activities/day-to-day instrumental activities, mobility, communication, humor/emotion, memory and thinking, and participation [47].

Medical Outcomes Short-Form Health Survey (SF-36): is a generic Health assessment tool. It consists of 36 questions, going over eight components, including functional capacity, physical aspects, pain, general health status, vitality, social aspects, emotional aspects, and mental health; evaluated by 35 questions and one more comparative question between the subject's current Health and that of a year ago [48].

Genetic Polymorphism Analysis: All individuals in the study will be investigated. The genetic polymorphisms of the ABO system gene (rs505922) and their possible association with stroke risk, two polymorphisms of the OPRM1 gene (rs1799971 and rs1799972) and their possible relationship with pain, and a polymorphism of the BDNF gene (rs6265) and its possible contribution to stroke risk, brain plasticity, and response to rehabilitation treatment in all investigated patients will be explored [49].
